# Computer-Assisted Differential Diagnosis of Pyoderma Gangrenosum and Venous Ulcers with Deep Neural Networks

**DOI:** 10.3390/jcm11237103

**Published:** 2022-11-30

**Authors:** Mattias Birkner, Julia Schalk, Peter von den Driesch, Erwin S. Schultz

**Affiliations:** 1 Institute of Medical Physics, Paracelsus Medical University Nuremberg, City Hospital of Nuremberg, 90419 Nürnberg, Germany; 2 Department of Dermatology, Paracelsus Medical University Nuremberg, City Hospital of Nuremberg, 90419 Nürnberg, Germany; 3 Department of Dermatology, Klinikum Stuttgart, Bad Cannstatt, 70174 Stuttgart, Germany

**Keywords:** pyoderma gangrenosum, leg ulcers, artificial intelligence, deep neural networks

## Abstract

(1) Background: Pyoderma gangrenosum (PG) is often situated on the lower legs, and the differentiation from conventional leg ulcers (LU) is a challenging task due to the lack of clear clinical diagnostic criteria. Because of the different therapy concepts, misdiagnosis or delayed diagnosis bears a great risk for patients. (2) Objective: to develop a deep convolutional neural network (CNN) capable of analysing wound photographs to facilitate the PG diagnosis for health professionals. (3) Methods: A CNN was trained with 422 expert-selected pictures of PG and LU. In a man vs. machine contest, 33 pictures of PG and 36 pictures of LU were presented for diagnosis to 18 dermatologists at two maximum care hospitals and to the CNN. The results were statistically evaluated in terms of sensitivity, specificity and accuracy for the CNN and for dermatologists with different experience levels. (4) Results: The CNN achieved a sensitivity of 97% (95% confidence interval (CI) 84.2–99.9%) and outperformed dermatologists, with a sensitivity of 72.7% (CI 54.4–86.7%) significantly (*p* < 0.03). However, dermatologists achieved a slightly higher specificity (88.9% vs. 83.3%). (5) Conclusions: For the first time, a deep neural network was demonstrated to be capable of diagnosing PG, solely on the basis of photographs, and with a greater sensitivity compared to that of dermatologists.

## 1. Introduction

Pyoderma gangrenosum (PG) is a rare but serious autoinflammatory neutrophilic dermatosis [1], showing an incidence of approximately 0.3–1/100,000, and mostly affecting women with a median disease onset at 60 years of age [2,3].

The disease typically starts with a sterile pustule (often after surgery or minimal trauma) that rapidly progresses, causing necrotising ulceration of variable depth and size with undermined violaceous wound borders. Most frequently, it occurs on the lower extremities, but other parts of the skin may also be affected. PG may be associated with either an inflammatory disease, such as inflammatory bowel disease or rheumatoid arthritis, or neoplastic diseases, such as haematological malignancy or solid tumours. Furthermore, retrospective cohort studies described a close association with paraproteinemia [4]. According to present knowledge, the pathogenesis is at least in part based on the enhanced activation of neutrophilic granulocytes, due to an aberrant activation of the inflammasome [5,6].

Treatment relies on immunosuppressive drugs, with the aim of slowing down the activation of neutrophilic granulocytes. In fact, the only approved treatment is the oral administration of corticosteroids [7]. Beyond this, cyclosporine and the TNF-alpha blocker infliximab demonstrated efficiency in randomised controlled trials [8,9]. In case of therapy resistance, there is an additional treatment approach that utilises intravenous immunoglobulins (IVIG), hitherto not proven in randomized studies [10].

In addition, efficient treatment with mycophenolate mofetil [11], azathioprine [12,13] and dapsone has been reported [14,15]. Most recently, the use of IL-1β-Inhibitor (canakinumab) and IL-1α-Inhibitor (anakinra), has been reported to be an effective therapy concept in some cases [16,17]. Since simple clear-cut diagnostic parameters are lacking, a combination of clinical and histological criteria was established in 1997 [1]. 

More recently, the so-called PARACELSUS-Score was developed to standardize diagnosis [18,19]. The PARACELSUS-Score consists of differently valued criteria, such as rapidly progressing disease, assessment of relevant differential diagnoses and a reddish-violaceous wound border. A total score value of 10 points or more indicates a high likelihood of PG and often correctly differentiates PG from venous leg ulcers [18,19].

Nonetheless, failed or delayed diagnosis is common [20,21] and represents a substantial risk for a worse clinical outcome. The differential diagnosis of conventional leg ulcers, which are mostly caused by a venous or arterial malfunction and may be associated with diabetes mellitus or arterial hypertension, is especially difficult [22]. For example, whereas in leg ulcers surgical debridement to induce granulation can be helpful, this intervention may cause disease progression in PG, eventually leading to amputation in the most severe cases [20]. Therefore, a prompt diagnosis and treatment are very important in PG [1] and health professionals should be aware of this disease. Supporting an early diagnosis of PG via the use of artificial intelligence might, therefore, be of substantial benefit for the patients.

In this paper, we present a deep neural network designed to support physicians and wound experts in diagnosing PG based on photographs of the wound. The application of deep learning techniques in healthcare has recently been a strong focus of computer vision researchers [23]. It has been successfully applied to assisted melanoma diagnosis and segmentation, and the analysis of dermatological wounds [24,25,26,27], but surprisingly not to the difficult and important task of identifying cases of pyoderma gangrenosum.

## 2. Materials and Methods

### 2.1. Data

Our dataset comprised 491 photographs of PG and conventional leg ulcers (most of which were due to proven venous insufficiency). The dataset was nearly balanced between the two diseases (PG:LU = 244:247). The patients were treated in the Klinikum Nuremberg and the Klinikum Stuttgart, both being large tertial referral centres for Dermatology between 2004 and 2021. The PG diagnosis was made in each case by the most experienced dermatologists (ES and PVDD), using the criteria of 1997, and all cases were additionally confirmed by the use of the recently described PARACELSUS-score [19] (≥10). After informed written consent was given, photographs were taken with high-quality consumer digital cameras that showed the entire wound. Each wound was only used once in our dataset. Only if multiple independent wounds on different extremities were present, was more than one photo of the same patient included in the dataset.

Out of this dataset, we randomly selected a balanced training dataset of 422 pictures (“dataset-422”) for the training of the convolutional neural network (CNN), and we put aside the remaining 69 pictures (balanced “validation set”) for final CNN validation and comparison with the dermatologists’ classification performances.

### 2.2. Performance Measures

In order to measure classification performance, we applied the following performance measures (where TP/FP (true/false positives) represent the numbers of correct/incorrect PG diagnoses, and TN/FN (true/false negatives) represent the numbers of correct/incorrect ulcus cruris (UC) diagnoses):

$$\mathrm{Accuracy}:\;\mathrm{rate}\;\mathrm{of}\;\mathrm{correct}\;\mathrm{diagnoses},\;ACC=\frac{\left(TP+TN\right)}{\left(TP+TN+FP+FN\right)}\;\mathrm{Sensitivity}:\;\mathrm{rate}\;\mathrm{of}\;\mathrm{correct}\;\mathrm{PG}\;\mathrm{diagnoses},\;SENS=\frac{TP}{\left(TP+FN\right)}\;\mathrm{Specificity}:\;\mathrm{rate}\;\mathrm{of}\;\mathrm{correct}\;\mathrm{UC}\;\mathrm{diagnoses},\;SPEC=\frac{TN}{\left(TN+FP\right)}$$

Furthermore, since the CNN yields probabilities for the conditions of PG and ulcus cruris (UC), and the decision threshold (cut-off) for one or the other diagnosis (50% throughout this work) can be manually varied, it is common to report the area under the curve (AUC) of the receiver operating characteristic (ROC; SENS over SPEC for varied decision threshold) [23].

### 2.3. Convolutional Neural Network (CNN) Training

Given the relatively small dataset, the implementation of a binary classification CNN based on transfer learning is the common approach, and has recently been successfully implemented in neural networks for melanoma classification [27,28,29,30,31,32] and wound assessment [33]. In this work, we decided to perform transfer trainings of common network architectures, such as InceptionV3, Resnet50, and VGG16, pretrained on millions of images [34] and openly accessible [35]. For our purpose, we did not freeze the pretrained networks’ filter weights, but allowed them to adjust for the purpose of wound image analysis.

Prior to the input into the neural network, all original megapixel images were resampled to 300 × 300 pixel resolution (nearest neighbour interpolation), i.e., the image aspect ratio was changed to 1:1.

### 2.4. Exhaustive Grid Search for Optimal Model Design

In order to achieve optimal performance, we followed the deep learning good practice and performed an exhaustive hyperparameter grid search, including nested k-fold cross-validations. The tested hyperparameters comprised e.g., the visual network type (Inception-V3, Resnet50, and VGG-16), the classifier network size, dropout rates, learning rate, and optimizer. For the exhaustive search, each of the 972 possible configurations was evaluated in a 3fold stratified cross-validation procedure, i.e., the training set of 422 pictures was randomly split into three balanced subsets of 140 pictures; then, the configuration was trained on any combination of two subsets of 280 pictures and evaluated on the remaining third subset. The average performance score ACC and AUC of the three runs yield realistic, non-optimistic estimates of a configuration’s performance on unseen data. 

The best performing model from a grid of 972 configurations was finally trained on the full dataset-422 and evaluated on the validation set.

To account for the relatively small number of training images, we applied the established technique of data augmentation, which multiplies the dataset by creating copies with arbitrary rotations, namely horizontal or vertical flips of images, without affecting the diagnosis. By doing so, the network learns that photographic perspective has no impact on the diagnosis and input information is spread across the entire visual input field. Throughout this work, we applied an augmentation factor of 4 (i.e., used 4 augmented copies).

### 2.5. Performance Validation

The optimal network’s performance was finally validated with the 69 unseen images from the validation set and the performance measures described above. In parallel, we compared the A.I.’s performance with the visual diagnoses made by the dermatologists on the same dataset, in an online survey.

## 3. Results

### 3.1. CNN Optimization

**Figure 1:**
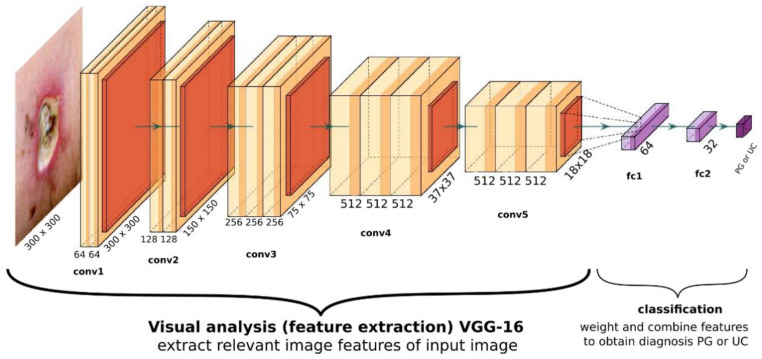
General overview of the optimal CNN’s structure. The visual part consists of a VGG16 architecture, with its final layer yielding 512 abstract features with size 18 × 18. The classifier consists of two fully connected dense layers with 64 and 32 neurons each, and a final two-neuron output yielding probability for PG and UC.

**Figure 2:**
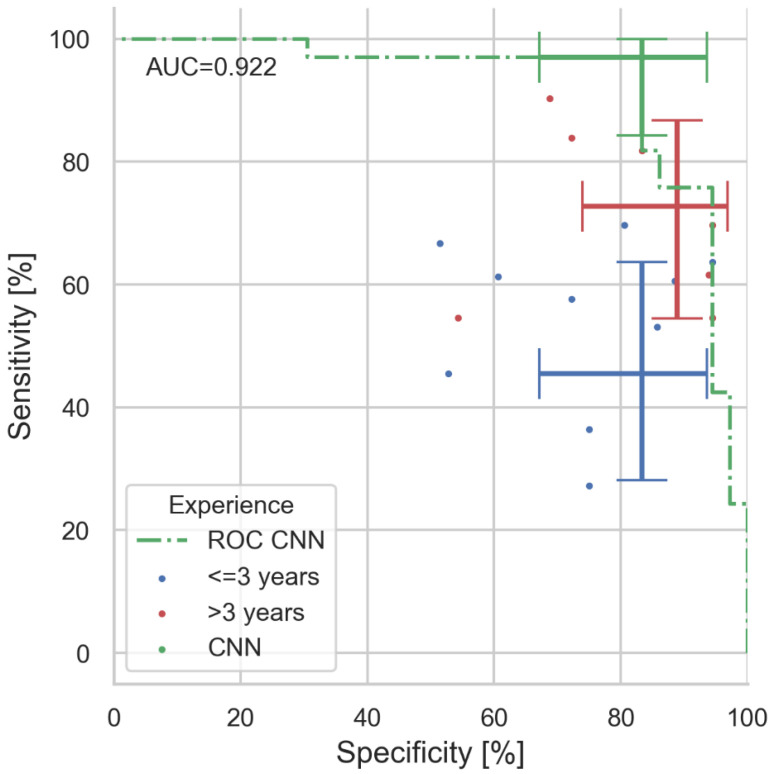
Results of man vs. machine validation. In total, 69 images (33 PG, 36 UC) were diagnosed by 18 dermatologists and our CNN algorithm. Coloured dots indicate the individuals’ performance (colour coded for professional experience). Error bars indicate the value and the 95% confidence intervals for sensitivity and specificity for CNN, expert, and juniors (majority decisions within experience group). With 97%, the CNN clearly outperforms even experienced physicians in sensitivity (correct PG diagnosis rate), whereas specificity is similar. The area under curve (AUC) of the presented CNN ROC is 0.922.

The optimal network performance was obtained with an architecture, which uses VGG16 visual processing, a 64-, 32-, and 2-neuron fully connected classification output (see Figure 1). When tested with the 69 unseen validation images, the network achieves an ROC curve with an area under curve (AUC) of 92.2% (see Figure 2).

### 3.2. Comparison with Human Specialists

For the final assessment of the AI’s performance, we conducted an anonymous online survey, in which 18 dermatologists and dermatological trainees of the Klinikum Nuremberg and the Klinikum Stuttgart centres were asked to make purely visual judgements of the 69 images in the validation set, without getting any further clinical information on the cases given. The participants were initially informed about the number of PG and UC cases included. Images presented to the physicians were the original images (i.e., not the resampled ones) adjusted to the browser window size. Numerical results are summarized in Table 1.

For clarity, we have grouped the participants into groups of professional experience: (1) all dermatologists, (2) experts with >3 years of experience and (3) juniors with ≤3 years of experience. Within each group, the majority determined the group’s final diagnosis for the image under test. The CNN achieves a sensitivity of 97% (CI: 84.2–99.9%) and surpass-es all dermatologists’ sensitivity of 72.7% (CI: 54.4–86.7%) significantly (McNemar’s *p* < 0.03), the experts’ sensitivity of 72.7% (CI: 54.4–86.7%; *p* < 0.03), and the juniors’ sensitivity of 45.5% (CI: 28.1–63.6%; *p* < 0.001). Differences in UC diagnosis (specificity) are not significant: with CNN: 83.3% (67.2–93.6%); all 88.9% (73.9–96.9%); experts: 88.9% (73.9–96.9%); and juniors: 83.3% (67.2–93.6%). 

All confidence intervals are given as the exact 95% binomial confidence interval (Clopper–Pearson). Graphical representation of the man–machine comparison on the receiver operating curve (ROC) are shown in Figure 2.

The CNN achieves an accuracy of 89.9% (80.2–95.8%), slightly better than all dermatologists and the experts with 81.2% (69.9–89.6%), and significantly better than juniors with 65.2% (52.8–76.3%, *p* < 0.001).

### 3.3. Visualizing the Network’s View of the Wound

**Figure 3:**
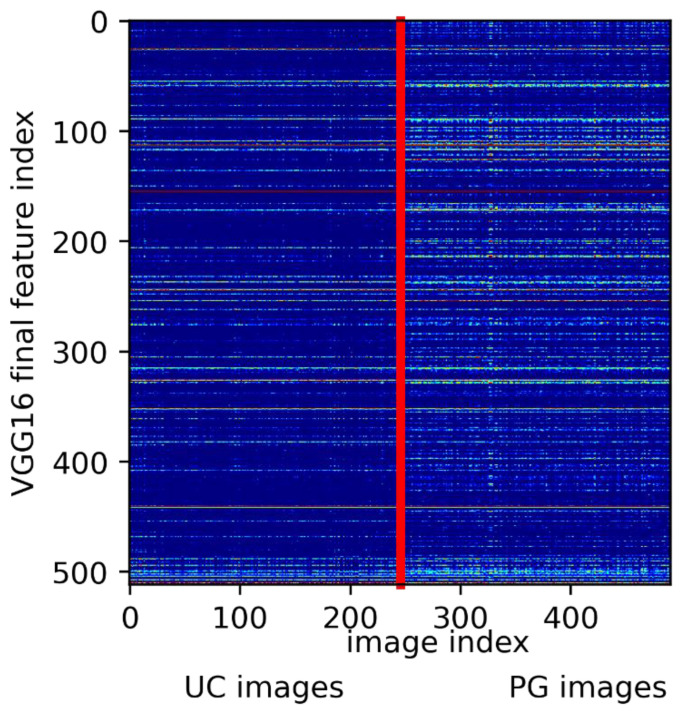
Extracted feature activations from the final VGG16 layer (*y*-axis) for all 491 images. Image indices (*x*-axis) were grouped in UC and PG ground truth. The vertical red line indicates the split between UC and PG diagnosis. Clearly, PG comes with the distinct expression of characteristic features, whereas UC shares features with PG.

As shown in Figure 1, the VGG-16 architecture consists of five consecutive convolutional blocks. It lies in the nature of this filtering process that, with increasing depth, the original image is down-sampled to smaller but deeper ‘features’. In its final stage our VGG-16 has down sampled the original image to 512 feature images of size 18 × 18. Each of these features represents a characteristic property of the input image that is deemed relevant for the network’s final diagnosis. The presence of a feature in a given image can be measured with its neurons’ activations (i.e., the mean brightness of the feature image). In Figure 3 we show the 512 activations of the VGG16’s final layer for all used images, sorted into UC and PG. Clearly, the ‘fingerprints’ of UC images are different from the PG fingerprints. UC images apparently have many common features with PG images, probably sharing the characteristics of a peripheral wound, whereas PG expresses an additional complex orchestration of hundreds of subfeatures. UCs being represented by a subset of PG features may lead the network to mistake a UC for a PG, as our network does occasionally. Note that activation levels of the 512 final features may eventually be used as a ‘distance measure’ to quantify how close images are in terms of PG appearance.

**Figure 4:**
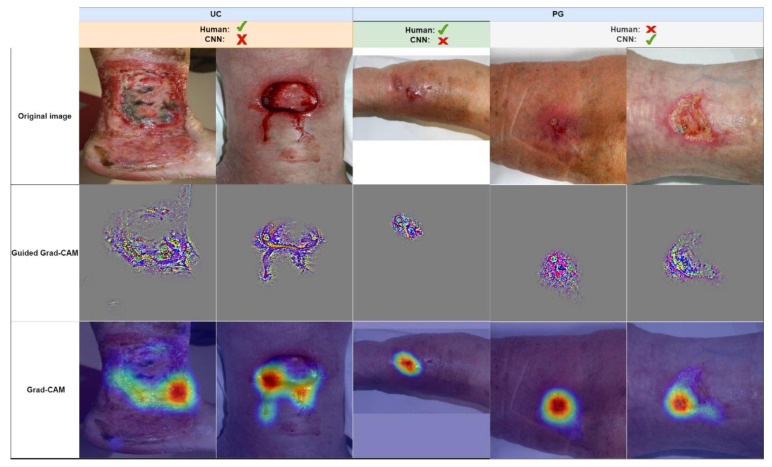
A few examples of images from the man vs. machine comparison, which were either misdiagnosed by humans or the network (correct majority diagnoses are indicated by a green checkmark, incorrect diagnoses by a red cross). Images are grouped in true diagnosis UC and PG. Guided Grad-CAM and Grad-CAM highlight the structures and areas that would lead the network towards a PG diagnosis. For the wrong UC interpretation (two leftmost columns), the CNN is misled by only small parts of the wounds, whereas for the correct PG diagnoses (two rightmost columns), the CNN accounts for the reddish area surrounding the lesion and the irregular wound border in the bottom left wound area.

Obviously, the algorithm has learned to visually differentiate between UC and PG during the training process. Luckily, with gradient-weighted class activation maps (Grad-CAM) [36], there are the means for a plausibility check of a CNN’s decision. Here, the data flow through the CNN is essentially reversed, from output to input, to highlight the areas of an input image that have the highest impact on the network’s final diagnosis. With guided Grad-CAM, one may even highlight the structures of the input image with the highest contribution to the output value. Examples are shown in Figure 4, where a few contradicting diagnoses from our man vs. machine comparison are shown.

## 4. Discussion

The aim of the present study was to develop a CNN to facilitate the diagnosis of PG for primary care physicians and wound managers, who are not routinely treating patients with this autoinflammatory disease. We made the first step towards this goal by developing a convolutional neural network (CNN), trained with 422 carefully validated images. The network showed a significantly higher sensitivity to diagnosing PG than dermatologists did (sensitivity 97% versus 72.7%), whereas the specificity of dermatologists was slightly better than that of the CNN (88.9% versus 83.3%). However, it must be mentioned that the diagnosis of PG does not only rely on the clinical image, as this may vary during the course of the disease, but often needs additional information, such as a search for associated diseases, histopathology, and laboratory examinations.

In an anonymous man vs. machine comparison, we demonstrated that sensitivity is clearly a matter of professional experience, as expected (see Figure 2). The authors hypothesize that this is due to the fact that, in case of doubts, juniors tend towards UC diagnoses due to the rare occurrence of PG in practice.

Our CNN tends to overestimate PG probability (expressed by the lower specificity); this is a welcome property, because the goal is to prevent the oversight of PG and to trigger further diagnostic procedures in dubious cases.

### 4.1. Outlook

Before initiating the inclusion of the A.I. in a routine clinical decision making process [37], further prospective evaluation and retraining with more images must be performed, including at further dermatological centres.

In addition, tools for visual plausibility checks must be developed and evaluated, to provide physicians with sufficient comprehensive information to accept or reject the machine’s proposal. As shown in the previous section and in Figure 3, visual feature fingerprints might be used to present the examiner with their/the images closest to the wound under investigation from the training set to support the network’s decision. In addition, (guided) Grad-CAM images may help the wound expert to understand whether the A.I. has taken the relevant wound areas into account.

Even though the PARACELSUS score [19] in part uses visual wound assessments for PG diagnosis, it is remarkable that the CNN outperformed its human contestants in sensitivity, solely by means of visual processing. It is therefore desirable, yet beyond the scope of this article, to understand which visual features cause the algorithm to decide upon one or the other diagnosis. Since features are extremely abstract and non-independent, the conversion of activation fingerprints into comprehensive visual interpretation is complex and subject to future research.

### 4.2. Limitations

The comparison of A.I. versus human experts’ visual diagnoses may be considered unfair, because in clinical practice, wound experts would consider additional non-visual information prior to making a diagnosis. Nonetheless, our results demonstrate that human experience greatly improves visual diagnosis and a visual AI tool may offer valuable, supplementary assistance for differentiating PG from leg ulcers.

Due to the rarity of PG, we were limited to a relatively small number of training images and validation images, compared to melanoma/nevi CNN developments [27,28,29,30,31,32] with typically thousands of training images and hundreds of validation images. Retraining the CNN with a larger dataset of validated images is likely to further improve the A.I.’s accuracy and, specifically, its specificity.

## Figures and Tables

**Table 1:** Statistical analysis from the man vs. machine comparison. For experience group analysis, the majority decided on the final diagnoses made per image, 95% confidence intervals are exact binomial Clopper–Pearson intervals, group size n is given in the header rows. Indicated *p*-values are McNemar’s significance levels for the difference to CNN performance (for non-significant differences with *p* > 0.1 ‘n.s.’ is given).

	CNN		Majority All Dermatologists (*n* = 18)			Majority Experts (>3 Year) (*n* = 8)			Majority Juniors (≤3 Year) (*n* = 10)		
	Result	95% CI	Result	95% CI	*p*-Value	Result	95% CI	*p*-Value	Result	95% CI	*p*-Value
**Sensitivity [%]**	97.0	84.2–99.9	72.7	54.4–86.7	<0.025	72.7	54.5–86.7	<0.025	45.5	28.1–63.6	<0.001
**Specificity [%]**	83.3	67.2–93.6	88.9	73.9–96.9	n.s.	88.9	73.9–96.9	n.s.	83.3	67.2–93.6	n.s.
**Accuracy [%]**	89.9	80.2–95.8	81.2	69.9–89.6	n.s.	81.2	69.9–89.6	n.s.	65.2	52.8–76.3	<0.001

## Data Availability

Not applicable.
